# Melatonin Attenuates PFOS‐Induced Reproductive Toxicity of Pregnant Mice due to Placental Damage Via Antioxidant, Anti‐Aging and Anti‐Inflammatory Pathways

**DOI:** 10.1002/bdr2.2423

**Published:** 2024-12-12

**Authors:** Jianqiu Han, Zhikai Lu, Yalei Qi, Tengfei Liu, Yongmei Li, Honghui Han, Chen Zhao, Xueyun Ma

**Affiliations:** ^1^ College of Ecological Technology and Engineering Shanghai Institute of Technology Shanghai China; ^2^ School of Life Sciences East China Normal University Shanghai China; ^3^ Molecular Neurogenetics Max Planck Institute of Psychiatry Munich Germany; ^4^ Renji Hospital, School of Medicine Shanghai Jiaotong University Shanghai China

**Keywords:** melatonin (MLT), perfluorooctane sulfonate (PFOS), placenta, pregnant mice, reproductive toxicity

## Abstract

**Background:**

Perfluorooctane sulfonate (PFOS), an industrially synthesized persistent organic pollutant (POP), is intricately intertwined with human production and daily life. It has been discovered that PFOS is related to an elevated incidence of birth defects in fetuses. In contrast, melatonin (MLT), a hormone secreted by the pineal gland, has been demonstrated to exert a protective effect on reproductive development.

**Methods:**

This paper investigates the protective effect of MLT against PFOS‐induced reproductive toxicity by simultaneously orally administering MLT to pregnant mice exposed to PFOS. The therapeutic effect was evaluated through the monitoring of pregnancy outcomes, histological changes in the placenta, apoptosis and proliferation of placental spongiotrophoblast, as well as the expression of antioxidant enzyme genes, anti‐aging genes, anti‐inflammatory genes and other relevant genes.

**Results:**

The results of the study demonstrated that MLT treatment reversed the adverse pregnancy outcomes caused by toxic PFOS, including a low number of implanted fetuses, low neonatal fetal weight, and an increased number of resorbed fetuses. MLT treatment decreased the levels of MDA, an oxidation product generated by PFOS in the placenta of pregnant mice, and increased the levels of the antioxidant enzyme SOD. Additionally, MLT was able to maintain the normalization of placental structure, reduce apoptosis and sustain the proliferation of placental spongiotrophoblast by upregulating the expression of antioxidant genes (Nrf2, CAT) and anti‐aging gene (Klotho), anti‐inflammatory gene (Hsd11b2), thereby counteracting the oxidative stress caused by PFOS in the placenta, moreover, it also reduced the expression of inflammatory genes (Pycard) in the placenta.

**Conclusions:**

The findings firmly establish the effectiveness of MLT in mitigating the harmful impacts of tainted PFOS on reproductive development during pregnancy. This provides a novel therapeutic approach for addressing PFOS‐induced birth defects in fetuses.

## 1 Introduction

Perfluorooctane sulfonate (PFOS) is a significant type of perfluorinated compound that has been extensively utilized in a multitude of applications, including coatings, polishes, adhesives, paper, umbrellas, and interior decoration materials over the past few decades (Hallberg et al. 2021). As a persistent organic pollutant that is difficult to break down, cable of long‐distance migration, and biomagnifies in the food chain, PFOS poses a serious threat to human health (Vassiliadou et al. 2015). High concentrations of PFOS have been detected in wildlife and humans, including in human blood and breast milk (Wei et al. 2021). Due to its toxicity and potential health effects, the use of PFOS has been gradually restricted in numerous fields (Qiu et al. 2024).

Due to its long half‐life and gradual metabolism within the human body, PFOS has the potential to accumulate in various organs, potentially leading to neurotoxicity, developmental toxicity, reproductive toxicity, and endocrine disruption (Chou and Lin 2020). For women, PFOS is associated with abnormal sex hormone levels and an increased risk of infertility and abnormal menstrual cycles (Shi et al. 2024). The report indicates that exposure to PFOS during pregnancy can cause placental dysfunction and fetal growth restriction (Aghaei et al. 2022). Previous studies have discovered that in utero exposure to PFOS impairs placental nutrient transfer and fetal growth, in part through reduced protein expression of the nutrient transporter protein SNAT4 and increased placental and fetal hepatic corticosterone levels (Wan et al. 2020). It has also been reported that PFOS exposure disrupts cytoskeletal dynamics, induces oocyte apoptosis associated with DNA damage, and promotes the overproduction of reactive oxygen species (ROS), which triggers mitochondria‐mediated apoptosis (Luo et al. 2018).

Experimental and preliminary epidemiological studies have established a link between PFOS exposure and oxidative stress based on systemic and cellular responses. For instance, PFOS blood levels disrupted redox‐related pathways and biomarkers in proteomic, metabolomic and lipidomic studies in adults, including pregnant women (Salihovic et al. 2020). Cross‐sectional studies have demonstrated a positive association between PFOS exposure and the circulating levels of inflammatory and pro‐oxidant markers in humans (Lu et al. 2019). Studies have reported higher immune cell counts and altered inflammatory responses in pregnant women, postpartum mothers, and children with environmental exposure to PFOS (Omoike et al. 2021). In laboratory settings, PFOS has been shown to generate ROS and induce inflammatory responses in vivo and in vitro (Jiao et al. 2021). In conclusion, a growing body of literature suggests that PFOS exposure promotes oxidative stress, which may be particularly detrimental to maternal and fetal health.

Melatonin (MLT), an indole amine primarily secreted and synthesized by the pineal gland through the hydroxylation of essential amino acids (Lee, Choi, and Back 2017), plays a role in regulating sleep patterns and circadian rhythms. Melatonin plays a role in regulating sleep patterns and circadian rhythms. Additionally, MLT has multiple functions, including anti‐tumor, sedative, immunoregulatory and reproductive effects (Ahmad et al. 2023). It may also regulate reproductive function by acting directly on the gonads, affecting the synthesis and secretion of sex hormones by directly targeting reproductive organs such as the ovaries (Yumnamcha et al. 2017). Moreover, MLT is a known potent antioxidant that acts as a free radical scavenger or metabolite that enhances antioxidant activity in cells (Galano, Tan, and Reiter 2013). Research has shown that MLT not only directly scavenges excess ROS produced in the body, but also stimulates antioxidant enzymes and inhibits the release of pro‐oxidant enzymes (Chen et al. 2020). Furthermore, as a lipophilic molecule, MLT can accumulate in high concentrations in mitochondria, making it a crucial factor in scavenging ROS from these organelles (Manchester et al. 2015). Consideration of MLT could prevent the damaging effects of nicotine and Cr(VI) on pregnant mice and their offspring (Ding et al. 2022; Liu et al. 2020). MLT may protect pregnant mice against PFOS‐induced reproductive toxicity. The objective of this study was to examine the potential of melatonin (MLT) to mitigate the reproductive effects of perfluorooctane sulfonic acid (PFOS) toxicity in pregnant mice, and to elucidate the underlying mechanisms.

## 2 Materials and Methods

### 2.1 Animals and Chemicals

Adult female ICR mice (8 weeks old, 28 ± 2 g) and adult male ICR mice (10 weeks old, 36 ± 2 g) were purchased from Shanghai Jihui Laboratory Animal Care Co. Ltd., they were housed in the Animal Experiment Center of East China Normal University. The animal feeding conditions were temperature 22°C ± 2°C, humidity 50%–60%, with a 12/12‐h light–dark cycle. The mice were provided with freely available food and water. All animals were domesticated for 1 week before the experiment. This study was performed according to the guidelines of the East China Normal University Animal Care and Use Committee. The study was approved by the East China Normal University Animal Care and Use Committee (AR2023‐067). in the Animal Experiment Center of East China Normal University at room temperature, humidity, and drinking water availability in accordance with the standard experimental animal feeding conditions with a 12/12‐h light–dark cycle.

MLT (purity, 98%, M5250) was purchased from Sigma‐Aldrich (St. Louis, MO, USA). Potassium perfluorooctane sulfonate (purity, 96.4%, DRE‐C15987122) was purchased from Dr. Ehrenstorfer (London, UK). Other unspecified chemicals were obtained from China National Pharmaceutical Group Corporation (Shanghai, China).

### 2.2 Animal Model Establishment and Grouping

The rationale for the experimental design was to administer both MLT and PFOS to pregnant mice and assess pregnancy outcome, placental histopathology, immunohistochemistry, and changes in antioxidant and anti‐inflammatory factors. Male and female mice were caged in a 1:2 ratio at 4:00 pm, and females were designated as gestationally 0.5 day pregnant (GD 0.5) in the morning when they were observed to be vaginally embolized (Tanner et al. 2018).

**FIGURE 1:**
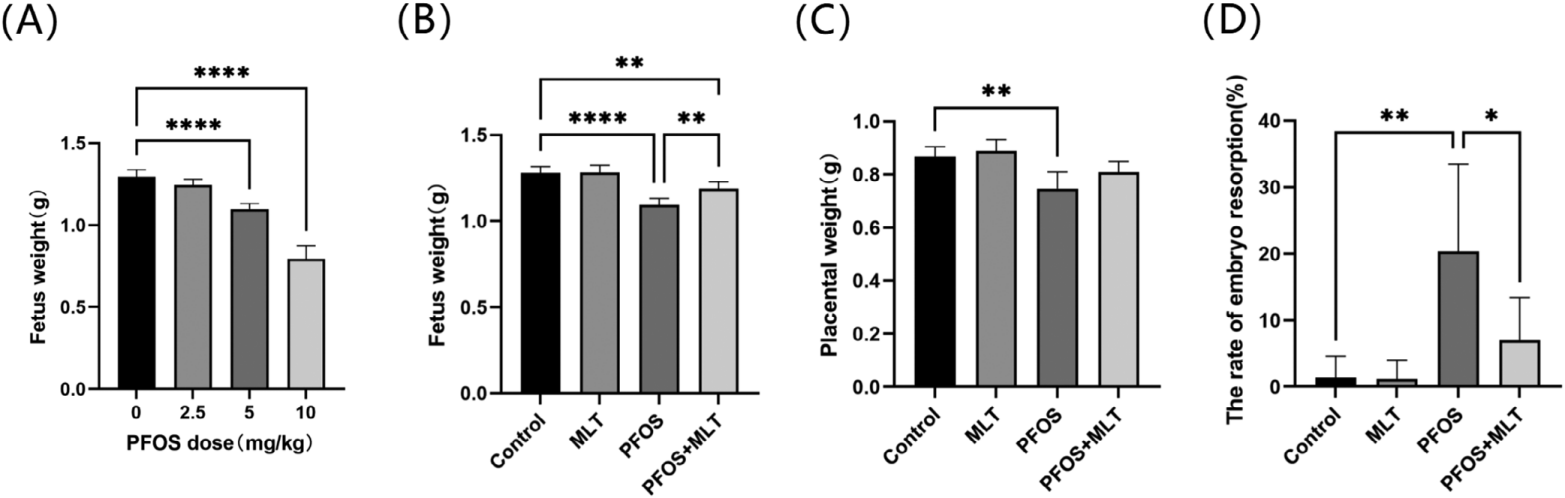
Gestational outcomes of experimental mice. (A) Fetal weights exposed to different doses of PFOS during pregnancy (*n* = 6); (B) Fetal weights of the four experimental groups (*n* = 6); (C) Weights of the placenta of the four experimental groups (*n* = 6); (D) The rate of fetal resorption (*n* = 6). Values are expressed as mean ± standard deviation. Statistical analyses were performed using one‐way ANOVA followed by Tukey's multiple comparison test. *p < 0.05; **p < 0.01; ****p < 0.0001.

In preliminary experiments, 24 pregnant mice were equally divided into four groups (*n* = 6). PFOS was dissolved in dimethyl sulfoxide (DMSO) (99.5% purity, SHBG6226V, Sigma‐Aldrich, USA) and then mixed with corn oil by gavage, and the final concentration of DMSO was controlled to be below 0.05%. From the day of fetus implantation (GD 4.5) to the eve of delivery (GD 17.5), mice were orally administered graded doses of 0, 2.5, 5 and 10 mg/kg PFOS, respectively. Based on the results of the preliminary experiments, the final dose of PFOS was determined to be 5 mg/kg/day. (Figure 1A).

Our previous study revealed that a dose of 10 mg/kg/day of MLT had a beneficial effect on reproduction in pregnant mice (Ding et al. 2022). Consequently, we employed this dose of 10 mg/kg/day in the current study. MLT was diluted with 0.9% saline. Twenty‐four pregnant mice were equally divided into four groups with each group consisting of six mice. The treatment group was administered PFOS (5 mg/kg/day) and MLT (10 mg/kg/day), the PFOS group was given PFOS (5 mg/kg/day), and the MLT group was administered MLT (10 mg/kg/day). The PFOS group received oral gavage, while the MLT group received intraperitoneal injection. Meanwhile, the control group was orally given the same dose of DMSO and corn oil.

### 2.3 Sample Collection

Pregnant mice were euthanized by cervical dislocation using carbon dioxide gas anesthesia followed by cesarean section on the 18.5th day of gestation. All live fetuses, resorbed fetuses, malformed fetuses, and placentas implanted in the uterus of the female mice were isolated, weighed, photographed, and classified for counting. Half of the treated placenta samples were then fixed with 4% paraformaldehyde and the other half was stored at −80°C.

The number of implanted embryos was determined by counting the embryonic nests on the endometrium. The resorption rate was calculated by dividing the number of resorbed fetuses by the number of implanted fetuses and multiplying the result by 100%.

### 2.4 Histopathological Analysis

The placentas were placed in paraformaldehyde and left overnight. After that, they were dehydrated in ethanol, cleared in xylene, and submerged in paraffin. All placental tissue was uniformly cut longitudinally, and 5‐μm‐thick tissue sections were prepared, the sections were then deparaffinized and rehydrated in graded ethanol. Hematoxylin and eosin staining were performed on the tissue sections before they were placed under the microscope (BX51; Olympus, Tokyo, Japan) for observation. Subsequently, the total area of the placenta, the area of the placental spongy trophoblastic layer, and the area of the placental labyrinth layer were measured using ImageJ software (v1.8.0).

### 2.5 Measurement of Antioxidant Enzyme Levels in Placenta

The placenta tissue was taken out from −80°C and placed into a mortar, a small amount of liquid nitrogen was added to facilitate grinding until it reached a fine powder consistency. Then the powder was collected and transferred into a new Eppendorf tube and a portion of the ground samples was extracted and diluted with an appropriate volume of pre‐cooled PBS buffer, followed by centrifugation at 5000g for 10 min. The resulting supernatant was transferred to a new Eppendorf tube for protein quantification using a microvolume spectrophotometer (DS‐11+, DeNovix, USA). The levels of lipid peroxidation were then assessed in accordance with the instructions provided with the MDA assay kit (S0131M, Beyotime, Shanghai) and the SOD assay kit (S0103, Beyotime, Shanghai).

### 2.6 Immunofluorescence Staining

Cleaved Caspase‐3 (CC‐3), Cleaved Caspase‐9 (CC‐9), and PCNA were analyzed by immunohistochemistry according to the manufacturer's instructions (Record Biological Technology Inc., China). Following deparaffinization and rehydration of placental sections, antigen extraction was conducted in 5% sodium citrate solution in a 100°C water bath for 20 min. After cooling, the sections were rinsed three times with PBST and antigenic site blocking was performed with 10% goat serum for 30 min. Subsequently, Primary antibodies CC‐3 (Asp 175, 1:300 dilution; Cell Signaling), CC‐9 (Asp 353, 1:200 dilution; Cell Signaling), and PCNA (PC 10, 1:500 dilution; Cell Signaling) were added and incubated at 4°C overnight. Then, the secondary antibody (Vectorlab, PK‐4001) was added, and the reaction was carried out for 30 min at room temperature and terminated with ABC reagent (Vectorlab, PK‐4001). After routine DAB (Vectorlab, SK‐4100) staining, sections were stained with hematoxylin and mounted. Images were acquired using a Leica AT2 scanner (Leica) and processed with Aperio Image Scope (Leica, v12.3.3). The stained samples were analyzed and quantified using ImageJ software(v1.8.0) to calculate the percentage of positive cells in all placental spongiotrophoblast cells in each group.

### 2.7 Total RNA Extraction and Quantitative Real‐Time PCR (RT‐qPCR)

Total RNA was extracted from placental samples according to the protocol provided by the TakaRa TRIZOL manufacturer, and the concentration and purity of the RNA were determined using a Thermo Scientific NanoDrop 2000C spectrophotometer. The RNA was then reverse transcribed using the PrimeScrpt RT kit with gDNA Eraser EasyScript (TaKaRa Clontech) for rapid cDNA synthesis. RT‐qPCR of the target genes was performed using the Applied Biosystems 7500 Real‐Time PCR System. The mRNA levels of nuclear factor erythroid 2‐related factor 2 (Nrf2), catalase (CAT), Klotho, PYD‐ and CARD‐containing structural domains (Pycard), and hydroxysteroid 11‐beta dehydrogenase 2 (Hsd11b2) were determined. The relative expression levels of the genes were calculated according to the 2^−ΔΔCt^ method, normalized to the housekeeping gene of β‐actin. Primer information is listed in Table 1.

**TABLE 1:** Primers for RT‐qPCR.

Gene name	GenBank number	Primer sequences (5′‐3′)		Product size (bp)
		Forward	Reverse	
Hsd11b2	NM_008289.2	TCCAAGGCAGCAATAGCACT	TCACATTAGTCACTGCATCTGTC	118
Pycard	NM_023258.4	ACTGTGCTTAGAGACATGGGC	TGGTCCACAAAGTGTCCTGTT	134
Klotho	NM_013823.2	TGACAACTACGTTCAAGTGGACA	CTTCTTGGCTACAACCCCGT	118
Nrf2	NM_001399226.1	TCTGCTGCAAGTAGCCTCG	TGGGCAACCATCACTCTGCT	115
CAT	NM_009804.2	ATGGTCACCGGCACATGAAT	GCCCTGGTCGGTCTTGTAAT	104
β‐Actin	NM_007393.5	CCAGCCTTCCTTCTTGGGTAT	GGGTGTAAAACGCAGCTCAG	374

### 2.8 Statistical Analysis

All data were statistically analyzed using GraphPad Prism 9.5 software. The results were expressed as mean ± standard deviation and statistical analysis was performed using one‐way analysis of variance (ANOVA) and Tukey's test for multiple comparisons. *p* < 0.05 was considered statistically significant and *p* < 0.01 was considered statistically very significant.

## 3 Results

### 3.1 MLT Alleviates Adverse Pregnancy Outcomes Induced by PFOS

As shown in Figure 1A, administering PFOS to pregnant mice led to a dose‐dependent reduction in fetal body weight. When dams were orally administered PFOS at a dose of up to 5 mg/kg/day, the fetal body weight decreased compared to that of fetuses in the control group (*p* < 0.01). When the PFOS dose was increased to 10 mg/kg/day, excessive weight loss was observed in the fetuses. Thus, a cautious decision was made to select 5 mg/kg/day as the final dose of PFOS for the experiment. To explore the protective effect of MLT against PFOS‐induced reproductive toxicity, pregnant mice were co‐administered with PFOS and MLT. Data on fetal body weight indicated that MLT attenuated PFOS‐induced loss of fetal weight (Figure 1B). The placental weight was significantly reduced in pregnant mice after PFOS induction (*p* < 0.01) and recovered following MLT treatment (Figure 1C). Statistical analysis showed that PFOS significantly increased fetal resorption (*p* < 0.01) (Figure 1D). The results suggest that MLT may effectively alleviate the adverse pregnancy outcomes induced by PFOS.

### 3.2 MLT Attenuates PFOS‐Induced Placental Damage

**FIGURE 2:**
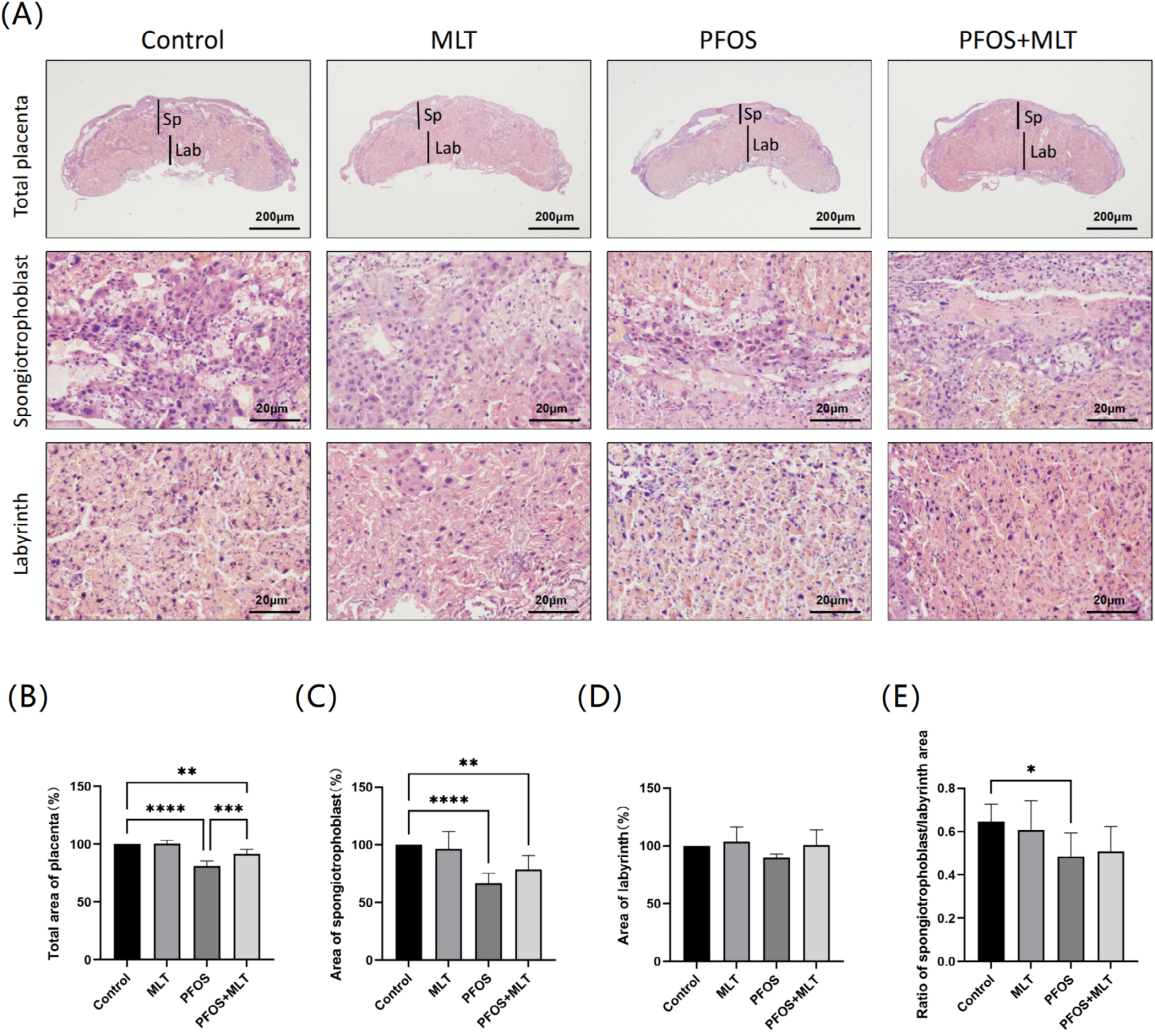
MLT attenuates PFOS damage to the placenta. (A) Representative stained images of the placenta at 2× and 20× magnification, with scale bars of 200 μm and 20 μm, respectively; (B) Total area of the placenta (*n* = 6); (C) Area of the placental spongiotrophoblast (*n* = 6); (D) Area of the labyrinth layer of the placenta (*n* = 6); (E) The ratio of the area of the placental trophoblastic layer to that of the connective layer (*n* = 6). Values are expressed as mean ± standard deviation. Statistical analyses were performed using one‐way ANOVA and Tukey's multiple comparison test. *p < 0.05; **p < 0.01; ****p < 0.0001.

Histopathological examination of placentas from pregnant mice revealed that MLT had a mitigating effect on PFOS‐induced reproductive toxicity by altering the area of placental structures (Figure 2A). Following PFOS induction, the total placental area and spongiotrophoblast area were significantly reduced compared to the Control group (*p* < 0.01) (Figure 2B,C). Additionally, the area of the placental labyrinth layer was also reduced after PFOS induction (Figure 2D). The ratio of the spongiotrophoblast area to the labyrinth area of the placenta after PFOS induction was significantly lower than that of the control group (*p* < 0.05) (Figure 2E). After MLT treatment, the total placental area, along with the area of the spongiotrophoblast and labyrinth layers, was partially restored.

### 3.3 MLT Mitigates Placental Oxidative Stress Induced by PFOS

**FIGURE 3:**
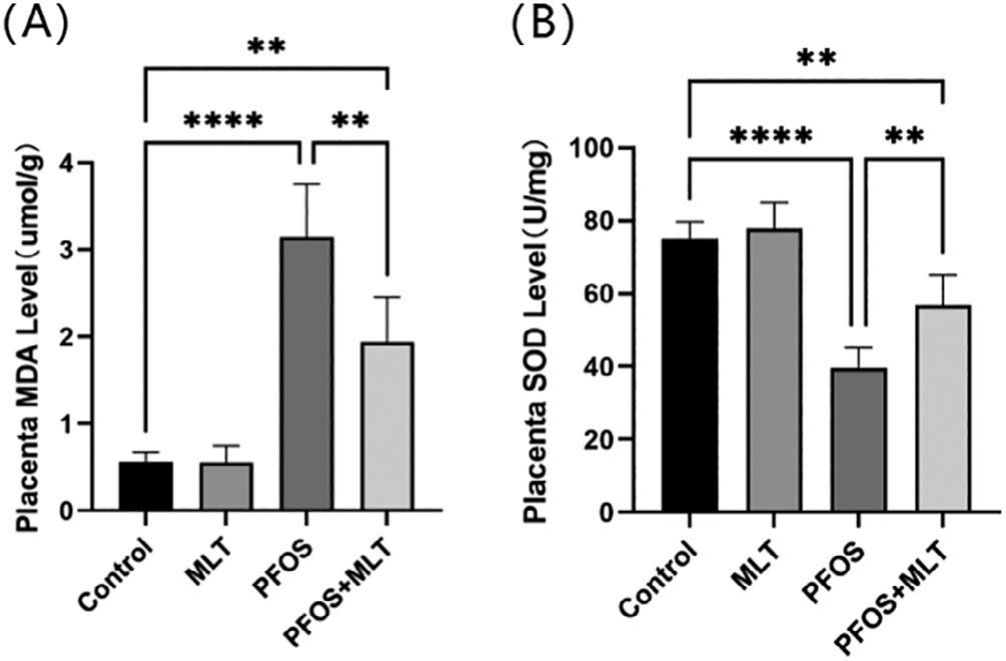
Accumulated MDA and SOD levels in the placenta of each group. (A) MDA levels in placental tissuer (*n* = 5); (B) SOD levels in placental tissue (*n* = 5). Data were expressed as mean ± standard deviation. Statistical analyses were performed using one‐way ANOVA and Tukey's multiple comparison test. **p < 0.01; ****p < 0.0001.

To determine the potential impacts of MLT on PFOS‐induced oxidative stress in the placenta, we measured the activity of antioxidant enzymes and the levels of oxidation products in the placentas of each group (Figure 3A,B). PFOS treatment notably increased the levels of the oxidation product MDA (*p* < 0.01) and decreased the levels of the antioxidant enzyme SOD in the placenta (*p* < 0.01), these adverse effects were reversed by MLT treatment. These results indicated that MLT attenuated PFOS‐induced oxidative stress in the placenta by reducing oxidation products and increasing the activity of antioxidant enzymes.

### 3.4 MLT Inhibits PFOS‐Induced Apoptosis in Placental Spongiotrophoblast

**FIGURE 4:**
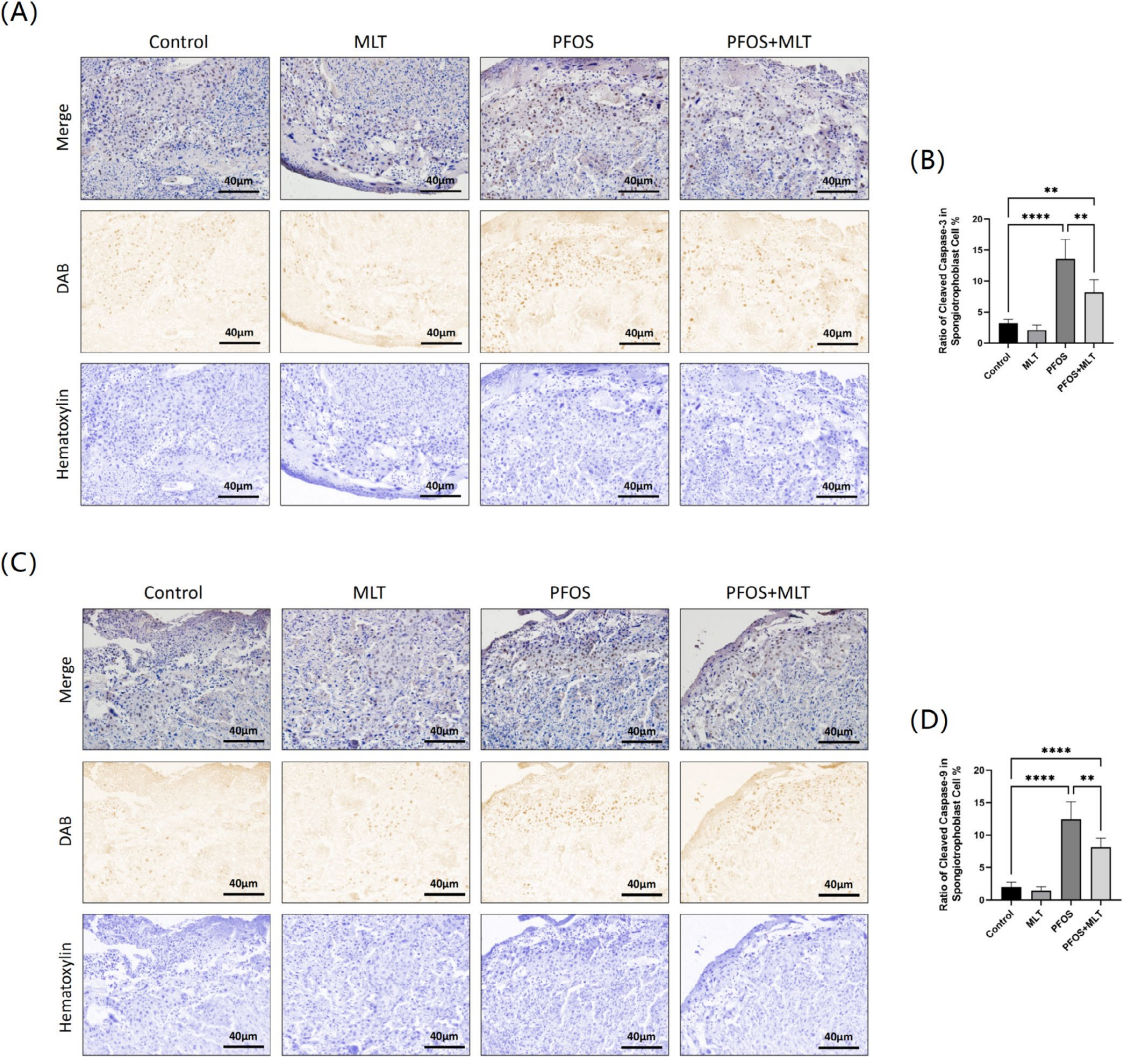
Expression levels of Cleaved Caspase3 and Cleaved Caspase9 in the placenta of each group. (A) Representative images of Cleaved Caspase3 immunostaining in placenta sections from the four experimental groups, magnified 20‐fold, with the scale lines representing 40 μm; (B) Ratio of Cleaved Caspase3‐positive placental spongitrophoblast cells to the number of total placental spongitrophoblast cells in the four experimental groups (*n* = 6); (C) Representative images of Cleaved Caspase9 immunostaining in placenta sections from the four experimental groups, magnified 20‐fold, with the scale lines representing 40 μm; (D) Ratio of Cleaved Caspase9‐positive placental spongitrophoblast cells to the number of total placental spongitrophoblast cells in the four experimental groups (*n* = 6). Data were expressed as mean ± standard deviation. Statistical analyses were performed using one‐way ANOVA and Tukey's multiple comparison test. **p < 0.01; ****p < 0.0001.

During placentation, the placental spongiotrophoblast invades the myometrium, endometrium, spiral arteries, and other tissues. They also provide nutritional support, immunity, and barrier protection for the development of the placenta (Chucri et al. 2010). Caspase is a marker of cell apoptosis, the results of the immunohistochemistry test showed a significant increase in the expression of the CC‐3 in the placenta of PFOS‐induced pregnant mice compared to that in the control mice (*p* < 0.01). When MLT was administered concurrently, the expression of CC‐3 showed a marked reduction (*p* < 0.01) (Figure 4A,B). Similar to the preceding results, CC‐9 expression was markedly elevated in the placental spongiotrophoblast following PFOS induction compared to the control group (*p* < 0.01) and exhibited a notable decline following MLT treatment (*p* < 0.01) (Figure 4C,D).

### 3.5 Effect of MLT on the Value‐Added Placental Spongiotrophoblast After PFOS Treatment

**FIGURE 5:**
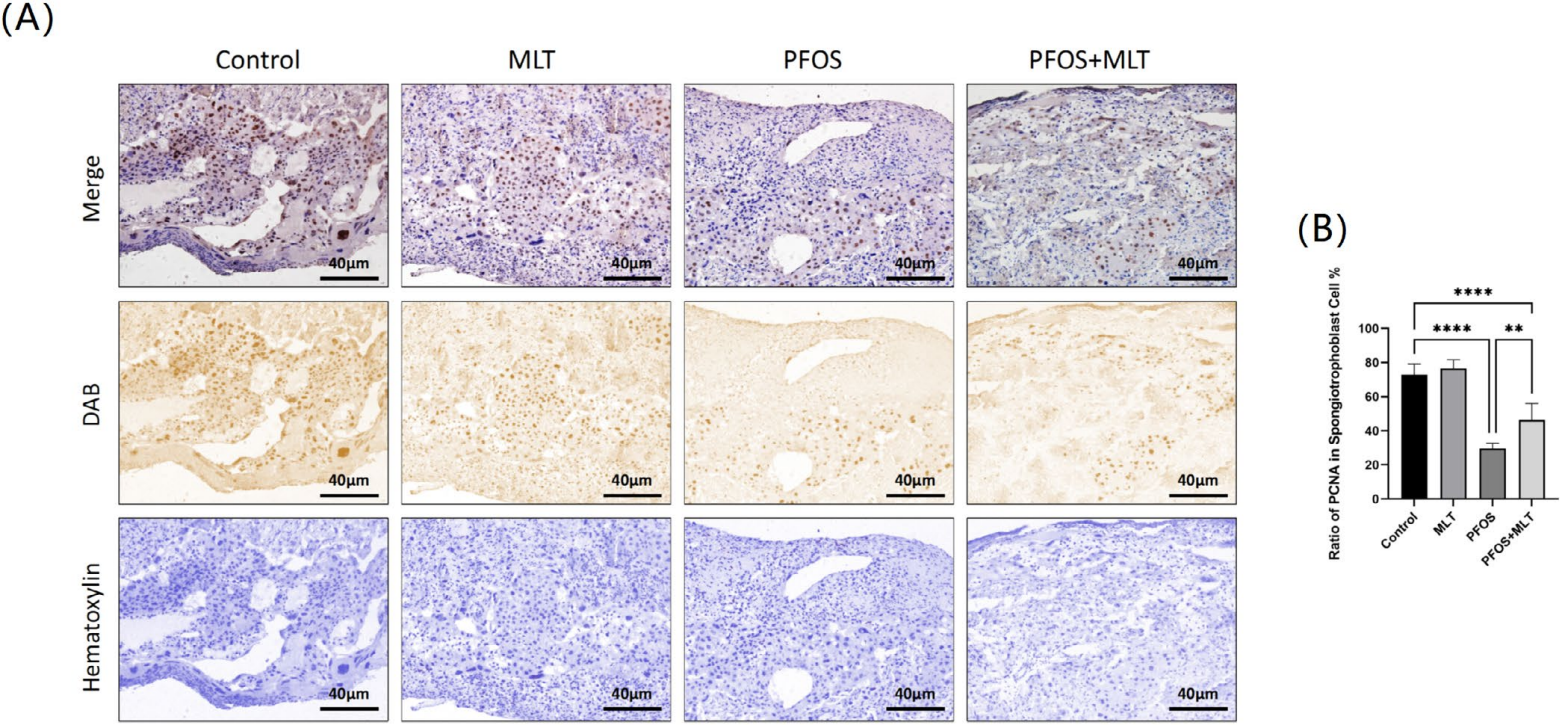
PCNA expression levels of placentas from each group. (A) Representative images of PCNA immunostaining of placenta sections from the four experimental groups, magnified 20‐fold, with scale lines representing 40 μm; (B) Ratio of PCNA‐positive placental spongitrophoblast cells to the number of total placental spongitrophoblast cells in the four experimental groups (*n* = 6). Data were expressed as mean ± standard deviation. Statistical analyses were performed using one‐way ANOVA and Tukey's multiple comparison test. **p < 0.01; ****p < 0.0001.

Placental spongiotrophoblast cells play a vital role during pregnancy. In the experiments, PCNA, acting as a marker of cell proliferation, was monitored by immunohistochemical assay (Figure 5A,B). The results showed that the expression of PCNA in the placenta of pregnant mice intoxicated by PFOS was significantly lower than that of the control group (*p* < 0.01), and the damage to the spongiotrophoblast cells was obvious. After administration of MLT, PCNA expression was restored to a certain extent (*p* < 0.01). This indicated that MLT could restrain the adverse effects of PFOS on the proliferation of placental spongiotrophoblast cells.

### 3.6 MLT Alleviated the Toxic Effects of PFOS by Regulating Placental Gene Expression in Pregnant Mice

**FIGURE 6:**
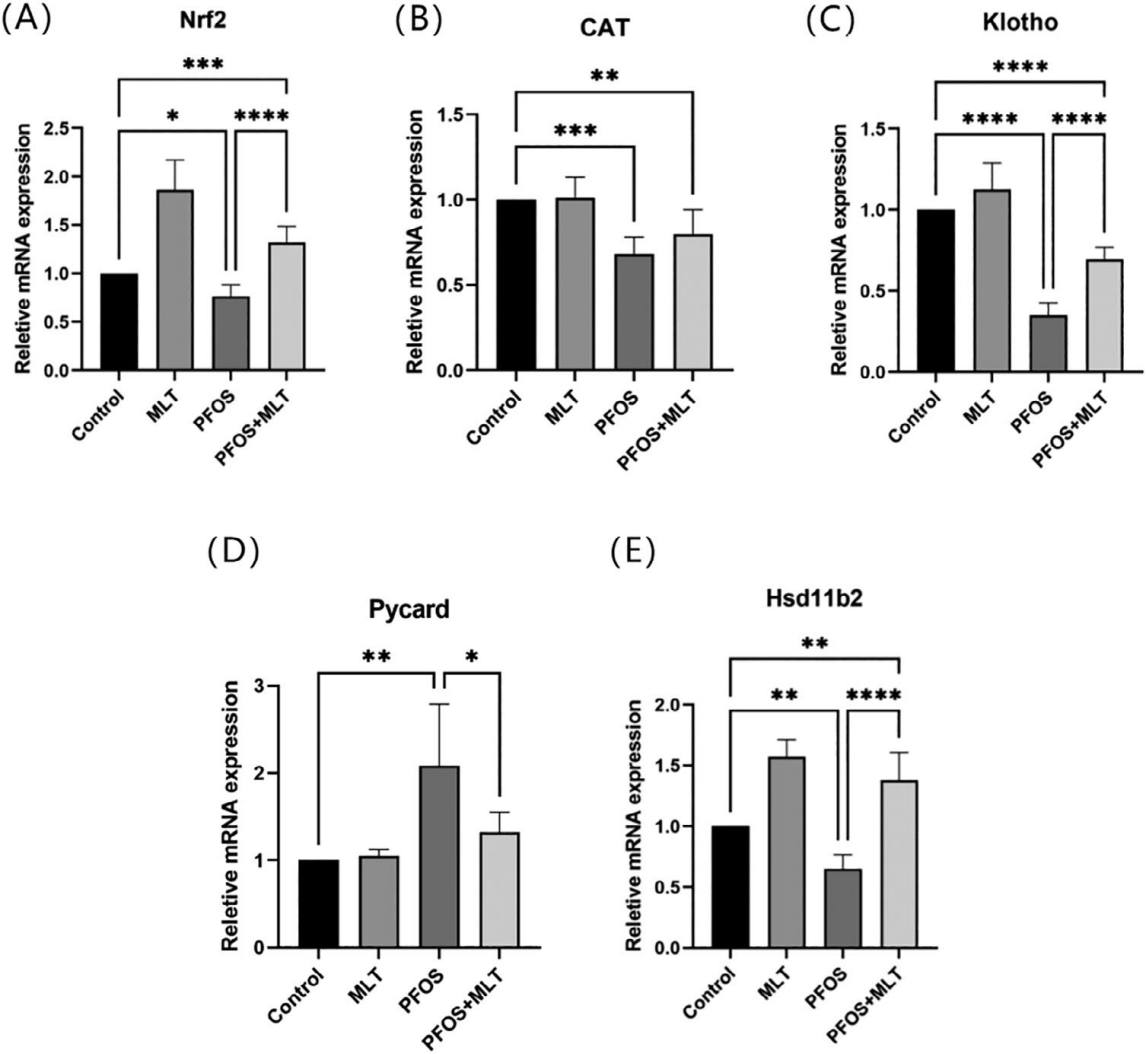
Placental gene expression levels detected by RT‐qPCR (n = 6). Data are expressed as mean ± standard deviation. Data were expressed as mean ± standard deviation. Statistical analyses were performed using one‐way ANOVA and Tukey's multiple comparison test. *p < 0.05; **p < 0.01; ***p < 0.001; ****p < 0.0001.

The expression of placental Nrf2, CAT, Klotho, Pycard and Hsd11b2 genes was examined by RT‐QPCR. The results indicated that the expression of antioxidant genes Nrf2, CAT and Klotho was significantly downregulated by PFOS compared to the control group (*p* < 0.05), however, treatment with MLT significantly reversed these results (*p* < 0.01) (Figure 6A–C). Additionally, overexpression of Pycard led to placental inflammatory infiltration. PFOS induction increased the expression of the pro‐inflammatory gene Pycard and simultaneously decreased the expression of the anti‐inflammatory gene Hsd11b2, The expression levels of both genes were reversed by MLT treatment (Figure 6D,E).

## 4 Discussion

Adverse effects of PFOS exposure on reproductive outcomes in pregnant mice are evident, as demonstrated by reduced fetal numbers, weight loss, and fetal resorption. Consistent with previous studies, high doses of PFOS decreased the total number of litters and increased the number of stillbirths (Narizzano et al. 2022). One potential explanation for this is that PFOS impairs the placental transfer of nutrients and fetal growth. Exogenous supplementation with MLT has been utilized in the treatment of various medical conditions, including psychiatric disorders, sleep disorders and metabolic disorders (Lim et al. 2022). It has also been found to play an important role in regulating maternal and fetal metabolic rhythms during pregnancy (McCarthy et al. 2019). The study showed that MLT alleviated PFOS‐induced adverse reproductive effects in pregnant mice. With the intervention of MLT, the pregnancy outcomes of reduced fetal weight and increased fetal resorption induced by PFOS were almost reversed to normal levels. Melatonin ameliorates maternal placental ischaemia, fetal cardiovascular damage and fetal neuroinflammation caused by oxidative stress in utero (Lee et al. 2019). MLT protects placental trophoblast cells from environmental stress and attenuates fetal growth restriction by inhibiting ROS‐mediated GCN2/ATF4/BNIP3‐dependent mitosis in placental trophoblast cells (Zhu et al. 2021).

The placenta is mainly composed of the fetal membranes, the spongiotrophoblast and the junction layer. Placenta formation occurs when the spongiotrophoblast invades the maternal meconium and myometrium during pregnancy. Under the influence of internal or external factors, the spongiotrophoblast may die out, thus hindering the exchange of nutrients and metabolism between the fetus and the mother (Groom and David 2018). Our study showed that PFOS staining reduced the total area of the placenta and the area of the spongy trophoblast, and these outcomes were reversed after MLT treatment. Apoptosis is a process of programmed cell death that removes unwanted or abnormal cells from the organism, and when disturbed by internal or external factors, apoptosis can lead to a number of diseases such as tumors and immune system disorders. The cysteine aspartase (Caspase) family plays an important role in the apoptotic process; Caspase‐3 is a marker for the initiation of the degradation phase of apoptosis and Caspase‐9 was a promoter of the apoptotic process. Apoptotic signals first activated the upstream initiator Caspase‐9 and then, through a cascade reaction, activated the downstream Caspase‐3, which ultimately caused nucleic acid breaks leading to apoptosis (Batoon et al. 2023; Kutscher and Shaham 2017). As shown in our results, PFOS upregulated the expression of CC‐3 and CC‐9 in placental spongiotrophoblast, thereby inducing their apoptosis. The quality of spongiotrophoblast was significantly improved by the administration of MLT. Numerous studies have shown that environmental pollutants inhibit PCNA expression in the placental spongiotrophoblast (Cao et al. 2023; Wang et al. 2020), leading to reduced female reproductive function.

PFOS affects reproductive function in mice by promoting oxidative stress and apoptosis induced by mitochondrial dysfunction (Wei et al. 2021). MDA is used to assess the level of oxidative stress as it is the end product of ROS attacking unsaturated fatty acids (Ito, Sono, and Ito 2019). In the experiment, a significant amount of MDA was detected in the PFOS group compared to the control group, while the antioxidant enzymes SOD was significantly decreased. Nrf2 is a redox‐sensitive transcription factor that regulates the expression of antioxidants including CAT (Liu et al. 2024). RT‐QPCR results showed that placental Nrf2 and CAT genes were significantly down‐regulated in PFOS‐induced pregnant mice. It was demonstrated that the expression levels of Nrf2 and CAT genes in the placenta of mice in the treatment group were significantly restored to higher levels; oxidative stress‐induced apoptosis of placental spongiotrophoblast associated with placental senescence was also reduced (Zhao et al. 2018). KIotho is an anti‐aging protein due to its antioxidant and anti‐inflammatory properties (Wang et al. 2023). Klotho, an aging gene is expressed in all types of trophoblast cells in the human placenta, The absence of Klotho may disrupt the normal physiological function of the placenta and lead to adverse birth outcomes (Chen et al. 2021). Moreover, Klotho‐mediated activation of the antioxidant Nrf2/ARE pathway was shown to influence apoptosis and senescence of placental spongiotrophoblast in hypoxic populations (Xu, Cheng, and Xue 2024). In the experiment, PFOS exposure led to a reduction in the expression level of the placental gene KIotho. However, the reduction was restored by intervention with MLT. It can thus be concluded that antioxidant and anti‐aging properties might be the mechanism through which MLT alleviated the toxic effects of PFOS in pregnant mice.

MLT has been shown to protect spongiotrophoblast from hypoxia/reoxygenation‐induced autophagy, inflammation and apoptosis (Sagrillo‐Fagundes et al. 2018). Apoptosis‐associated speck‐like protein (Pycard) inhibits cell proliferation and triggers inflammation by inducing apoptosis through the activation of the caspase pathway (Chi et al. 2020). Our study found that MLT treatment significantly reduced PFOS‐induced upregulation of Pycard to normal levels. Prenatal stress exposes the fetus to excessive glucocorticoids during pregnancy, resulting in abnormal body weight. Hsd11b2, which is produced in placental syncytiotrophoblast cells, converts glucocorticoids into an inactive form to safeguard normal fetal development (Togher et al. 2014). Steroid hormones exert a significant anti‐inflammatory function within the body. Environmental pollutants could inhibit the expression of the steroidogenic gene (Hsd11b2), which in turn affects the synthesis of steroid hormones (Zhang et al. 2018). Previous studies have shown that exposure to other PFAS‐like substances during pregnancy in pregnant mice can also lead to impaired placental function, causing gestational hypertension and neonatal death, as well as altering the expression of genes involved in pathways critical to placental function, causing immune and inflammatory responses (Bangma et al. 2020; Blake and Fenton 2020; Chambers, Hopkins, and Richards 2021). According to our results, PFOS significantly downregulated the expression level of Hsd11b2 in the placenta, and MLT treatment increased its expression. It can be concluded that anti‐inflammatory may be one of the mechanisms by which MLT alleviates the toxic effects of PFOS in pregnant mice.

## 5 Conclusions

Studies have shown that MLT treatment mitigates PFOS‐induced adverse pregnancy outcomes and safeguards normal fetal development. Moreover, MLT can preserve the normal structure of the placenta, restrain PFOS‐induced apoptosis of placental spongiotrophoblast, and promote their proliferation. Additionally, MLT exhibits anti‐inflammatory, anti‐aging and antioxidant effects, capable of reducing PFOS‐induced oxidative stress and inflammation, thereby maintaining the normal physiological function of the placenta. In conclusion, the results of this study suggest that MLT may be an effective therapeutic measure for the treatment of PFOS‐induced female reproductive injury. This provides a novel approach to the treatment of neonatal birth defects caused by environmental pollutants.

## Author Contributions

**Jianqiu Han:** investigation, data curation, writing – original draft preparation. **Zhikai Lu:** investigation, data curation, writing – original draft preparation. **Yalei Qi:** investigation, data curation. **Tengfei Liu:** investigation, validation. **Yongmei Li:** investigation, validation. **Honghui Han:** supervision, writing – reviewing and editing. **Chen Zhao:** conceptualization, supervision and writing – reviewing. **Xueyun Ma:** conceptualization, data curation, writing – reviewing and editing.

## Conflicts of Interest

The authors declare no conflicts of interest.

## Data Availability

The data that support the findings of this study are available on request from the corresponding author. The data are not publicly available due to privacy or ethical restrictions.
